# Burdens, resources, health and wellbeing of nurses working in general and specialised palliative care in Germany – results of a nationwide cross-sectional survey study

**DOI:** 10.1186/s12912-021-00687-z

**Published:** 2021-09-06

**Authors:** Elisabeth Diehl, Sandra Rieger, Stephan Letzel, Anja Schablon, Albert Nienhaus, Luis Carlos Escobar Pinzon, Pavel Dietz

**Affiliations:** 1 Institute of Occupational, Social and Environmental Medicine, University Medical Center of the Johannes Gutenberg University Mainz, Obere Zahlbacher Str. 67, 55131 Mainz, Germany; 2 Institute for Health Services Research in Dermatology and Nursing (IVDP), University Medical Centre Hamburg-Eppendorf, Building W38, Martinistraße 52, 20246 Hamburg, Germany; 3 Department for Occupational Medicine, Hazardous Substances and Health Science, Institution for Accident Insurance and Prevention in the Health and Welfare Services (BGW), Pappelallee 33/35/37, 22089 Hamburg, Germany; 4 Federal Institute for Occupational Safety and Health (BAuA), Nöldnerstr. 40-42, 10317 Berlin, Germany

**Keywords:** Stress, Strain, Burnout, Depression, Intention to leave the profession, Prevention

## Abstract

**Background:**

Palliative care in Germany is divided into general (GPC) and specialised palliative care (SPC). Although palliative care will become more important in the care sector in future, there is a large knowledge gab, especially with regard to GPC. The aim of this study was to identify and compare the burdens, resources, health and wellbeing of nurses working in GPC and SPC. Such information will be helpful for developing prevention programs in order to reduce burdens and to strengthen resources of nurses.

**Methods:**

In 2017, a nationwide cross-sectional survey was conducted. In total, 437 nurses in GPC and 1316 nurses in SPC completed a questionnaire containing parts of standardised instruments, which included parts of the Copenhagen Psychosocial Questionnaire (COPSOQ), the Patient Health Questionnaire (PHQ-2), the Resilience Scale (RS-13) Questionnaire, a single question about back pain from the health survey conducted by the Robert Koch Institute as well as self-developed questions. The differences in the variables between GPC and SPC nurses were compared.

**Results:**

SPC nurses reported higher emotional demands as well as higher burdens due to nursing care and the care of relatives while GPC nurses stated higher quantitative demands, i.e. higher workload. SPC nurses more often reported organisational and social resources that were helpful in dealing with the demands of their work. Regarding health, GPC nurses stated a poorer health status and reported chronic back pain as well as a major depressive disorder more frequently than SPC nurses. Furthermore, GPC nurses reported a higher intention to leave the profession compared to SPC nurses.

**Conclusions:**

The findings of the present study indicate that SPC could be reviewed as the best practice example for nursing care in Germany. The results may be used for developing target group specific prevention programs for improving health and wellbeing of nurses taking the differences between GPC and SPC into account. Finally, interventional and longitudinal studies should be conducted in future to determine causality in the relationship of burdens, resources, health and wellbeing.

**Supplementary Information:**

The online version contains supplementary material available at 10.1186/s12912-021-00687-z.

## Background

Due to demographic changes, western European societies are faced with numerous challenges and changes. Higher life expectancy, in particular in older age groups, is related to more patients with incurable and life-threatening diseases [1]. The Federal Statistical Office in Germany predicts an increase of persons being in need of care from 3.4 million in 2017 up to 4.1 million in 2030, and to 5.4 million in 2050 [2]. In the past, primarily cancer patients have benefitted from palliative care, but today and in future, people with non-oncological diseases, multimorbid patients [3] and patients suffering from dementia [4] should also benefit from palliative care. Over the course of these developments, palliative care will become more important in the care sector.

The World Health Organization (WHO) defines palliative care as “an approach that improves the quality of life of patients and their families facing the problem associated with life-threatening illness, through the prevention and relief of suffering by means of early identification and impeccable assessment and treatment of pain and other problems, physical, psychosocial and spiritual” [5]. The implementation of palliative care in Europe differs widely, as the European Association for Palliative Care (EAPC) Atlas of Palliative Care in Europe 2019 demonstrates [6]. In Germany, palliative care is divided into general palliative care (GPC), sometimes also called general outpatient palliative care, and specialised palliative care (SPC). The majority of patients are treated within GPC in outpatient care, in nursing homes or in hospitals within the contractual healthcare system [7]. According to the German Society for Palliative Medicine (DGP), around 90% of approximately 850.000 dying people in Germany are in need of palliative care, but only 10% of them are in need of SPC [8, 9]. SPC is for dying people who need a particularly complex treatment and the medical support of them is more demanding, such as a complex pain management. SPC includes the specialist outpatient palliative care (SAPV), the inpatient hospices or palliative care units in hospitals and is conducted through interdisciplinary teams (see additional Table 1). Palliative care should be made available at different levels, so that the aims and objectives of all institutions are different. Palliative care units for example should improve or stabilise the condition of individual patients in order to discharge them, if possible, to their own homes. SAPV-teams in contrast should enable a dignified death in familiar surroundings. Moreover, the ‘Charter for the Care of the Critically Ill and the Dying’ in Germany published by the German Association for Palliative Medicine (DGP), the German Hospice and Palliative Care Association (DHPV) and the German Medical Association (BÄK) formulated recommendations as the basis for a national strategy. Dying, death and grief are part of life and all human beings have a right to a dignified death. Further, all critically ill and dying people have a right to

- comprehensive medical, nursing, psychosocial and spiritual care that takes into account their individual situation and palliative/hospice care needs.
- appropriate, qualified and, if required, multiprofessional care.
- care based on best practice.
- benefit from care that takes into account internationally recognised and adopted recommendations and standards regarding the delivery of palliative care [10].

Further information on palliative care in Germany can be found in the statement of the German National Academy of Sciences Leopoldina and the Union of German Academies of Sciences and Humanities from 2015 [11] and the EAPC Atlas of Palliative Care in Europe 2019 [6].

Numerous international studies have shown that palliative care is demanding [12]. For example, organisational framework conditions such as many administrative tasks [13] or insufficient personnel to handle workloads [14], quantitative demands such as time pressure [15], demands resulting from nursing care such as therapy-resistant pain or lifting and carrying of patients [16], and in particular confrontation with illness, suffering and death of patients and their families showed to be demanding [13, 15, 17, 18]. Nevertheless, studies do not report higher levels of stress or demands of palliative care nurses compared to nurses in other disciplines [15, 17]. Within an extensive literature review, Mary Vachon (1995) summarized that only the first early studies in the field of palliative care observed higher stress levels of palliative care nurses, but later studies did not. She hypothesised that the early recognition of the potential stress in the field of palliative care lead to the development of appropriate organisational and personal coping strategies to deal with the stressors of this field [19]. According to previous studies, palliative care nurses seem to have a wide range of resources. For example, organisational resources such as the meaningfulness of work [16, 20] or supervision [18] as well as social resources such as the team, were reported to be very important resources [18, 21, 22]. In addition, personal resources like resilience [23, 24], humour, self-care [22, 25, 26], hobbies [21], physical activity [27], spirituality [21, 22] or empathy [18], a special personality [28] and sociodemographic factors like age and professional experience [19] might help to cope with work demands and promote nurses’ health. Overall, it seems that palliative care nurses are satisfied with their work [27, 29, 30] and report low levels of burnout [13, 15, 17, 31]. In contrast, studies outside the palliative care setting reported consistently of an increasing workload with high burdens and a high intention to leave the profession of nurses [32–34]. Further, a recent literature review also suggests that healthcare professionals in GPC experience more symptoms of burnout than those in SPC settings [35].

Healthy and satisfied nurses are of enormous importance worldwide, because their health may have an effect on the quality of the services offered by the health care system [36]. Studies from Germany investigating palliative care aspects exclusively refer on SPC [16, 18, 37, 38]. For example, with focus on the resource ‘team’, Müller et al. (2009, 2010) reported that the team itself was ranked as the most important protective factor of nurses working in hospices [38] and palliative care units [18]. This finding was confirmed by Diehl et al. (2018) for SAPV-teams, inpatient hospices and palliative care units in hospitals [30]. Gencer et al. (2019) compared the working conditions, such as the overall stress level of nurses working in palliative care units and SAPV-teams, showing that, for example, the stress level is higher for nurses in palliative care units [37].

To the best of our knowledge, a study comparing the burdens, resources, health and wellbeing of nurses in GPC and SPC in Germany has not been performed so far. Therefore, the aim of the present study was to address this gap by identifying and comparing the burdens, resources, health and wellbeing of nurses in GPC and SPC in Germany. This information may be relevant and could be used for developing target group specific prevention programs in order to reduce burdens and to strengthen resources of nurses in palliative care. Furthermore, a comparison of the working situation of GPC and SPC nurses may contribute to new findings, which could have relevant implications for developing interventional studies, with the goal of improving the health status of nurses and enhancing job satisfaction.

## Methods

### Study design

A nationwide cross-sectional empirical study was conducted in 2017. Ethical approval to perform the study was obtained by the ethics committee of the State Chamber of Medicine in Rhineland-Palatinate (Clearance number 837.326.16 (10645)).

Data among nurses of GPC were collected from a 10% sample (3278: 1190 nursing homes, 1961 outpatient care, 127 hospitals) of the database from the Institution for Statutory Accident Insurance and Prevention in Health and Welfare Services in Germany. Due to data protection rules, this institution communicated with the health facilities of which 126 (3.8%) had agreed to participate in the survey.

Because there is no national register for nurses working in SPC nor specialised palliative care institutions, first, all medical facilities in the specialised palliative care were identified (950: 358 SAPV institutions, 343 palliative care units, 249 inpatient hospices) by an internet search. Secondly, an institution-related sample was drawn. Out of 532 institutions in the sample, 246 were willing to participate (46.2%).

As described, the present study focused firstly on medical facilities. The participating health facilities of GPC and SPC informed the study team about the number of nurses (nurse, geriatric nurse, nursing assistant or nurse in training and carrying for patients) and if they preferred to answer a paper-and-pencil (with a franked return envelope) or an online-questionnaire (with an access code). The participation was voluntary and anonymous. Table 1 provides information about the amount of questionnaires send to the different health facilities in GPC and SPC.

**Table 1:** Number of questionnaires send out to the health facilities and response rates

Question-naires send out	GPC									SPC								
	Outpatient care			Hospitals			Nursing homes			SAPVs			Hospices			Palliative care units		
	Send	Return	Rate (%)	Send	Return	Rate (%)	Send	Return	Rate (%)	Send	Return	Rate (%)	Send	Return	Rate (%)	Send	Return	Rate (%)
Paper	327	80	24.5	160	29	18.1	1777	315	17.7	749	254	33.9	1160	500	43.1	864	405	46.9
Online	329	16	4.9	0			389	31	8.0	429	88	20.5	206	45	21.8	131	34	26.0
Total	656	96	14.6	160	29	18.1	2166	346	16.0	1178	342	29.0	1366	545	39.9	995	439	44.1
	Send			Return*			Rate (%)			Send			Return**			Rate (%)		
Total	Paper: 2264			Paper: 445			Paper: 19.7			Paper: 2773			Paper: 1171			Paper: 42.2		
	Online: 718			Online: 52			Online: 7.2			Online: 766			Online: 200			Online: 26.1		
	Total: 2982			Total: 497			Total: 16.7			Total: 3539			Total: 1371			Total: 38.7		

*Note.* Rate = response rate, *26 questionnaires (21 x paper-and-pencil, 5 x online) no identification to type of institution possible, **45 questionnaires (12 x paper-and-pencil, 33 x online) no identification to type of institution possible

### Questionnaire

The questionnaire addressed four major issues. I) Basic sociodemographic characteristics (gender, age, etc.) and characteristics on current profession. II) Questions about occupational burden, III) questions on organisational, social and personal resources, and IV) questions concerning health and well-being. Since the specific job-related conditions between GPC and SPC are somewhat different, some questions were slightly adapted. The questionnaire contained questions from standardised, reliable and valid instruments:

- Copenhagen Psychosocial Questionnaire (COPSOQ)

Parts of the German standard version of the COPSOQ version II [39] were used. The COPSOQ is a valid and reliable questionnaire to assess psychosocial work environmental factors and health in the workplace [40]. The subscales used for the present study were ‘quantitative demands’, ‘emotional demands’, ‘demands for hiding emotions’, ‘meaning of work’, ‘workplace commitment’, ‘satisfaction with life’, ‘self-rated health’, ‘burnout’ and ‘intention to leave the profession’. The COPSOQ scales mostly consisted of several items and were collected with a five-point Likert scale (categories ranging from e.g. never to always). The ‘satisfaction with life’ scale was collected with a 7-point Likert scale (categories ranging from do not agree at all to fully agree) and the ‘self-rated health’ scale as well as the ‘intention to leave the profession’ scale were collected with a single question (Table 2). The single items of the COPSOQ scales were transformed to a theoretical range from 0 (the lowest possible amount of the aspect under investigation) to 100 (the highest possible value). The transformation of the categories into point values is a standardised procedure and was also used in the German validation study [40].

- Patient Health Questionnaire-2 (PHQ-2)

**Table 2:** Sources and variables of the questionnaire

	Source	Number of items	Example of items	Outcomes of variables
**Burdens**				
Burden due to organisational framework conditions	pilot study	7	*How strong is the burden due to the care of to many patients?*	scale 0–100, high = negative
Quantitative demands	COPSOQ	4	*Do you have to work very fast?*	scale 0–100, high = negative
Emotional demands	COPSOQ	3	*Is your work emotionally demanding?*	scale 0–100, high = negative
Demands for hiding emotions	COPSOQ	3	*Does your work require that you hide your feelings?*	scale 0–100, high = negative
Emotional burden due to death	pilot study	9	*How strong is the burden due to patients dying a painful death?*	scale 0–100, high = negative
Burden due to care of patients	pilot study	6	*How strong is the burden due to depressive patients?*	scale 0–100, high = negative
Burden due to nursing care	pilot study	5	*How strong is the burden due to lifting and carrying of patients?*	scale 0–100, high = negative
Burden due to care of relatives	pilot study	12	*How strong is the burden due to relatives cause unrest?*	scale 0–100, high = negative
**Resources**				
*Organisational*				
Meaning of work	COPSOQ	3	*Do you feel that the work you do is important?*	scale 0–100, high = positive
Workplace commitment	COPSOQ	4	*Do you enjoy telling others about your place of work?*	scale 0–100, high = positive
Meaningfulness of work	pilot study	1	*How much do the following help you to handle the workload?*	not/little helpful vs. quite/very helpful
Gratitude of patients/relatives	pilot study	each case 1	*How much do the following help you to handle the workload*	not/little helpful vs. quite/very helpful
Recognition through supervisor/ colleagues/ patients/relatives/ social context/ salary	pilot study	each case 1	*Do you receive recognition for your work from …*?	disagree/slightly disagree vs. slightly agree, agree
*Social*				
Good working team	pilot study	4	*I get help and support from colleagues in emergencies.*	scale 0–100, high = positive
Family	pilot study	1	*How much do the following help you to handle the workload?*	not/little helpful vs. quite/very helpful
Friends	pilot study	1	*How much do the following help you to handle the workload?*	not/little helpful vs. quite/very helpful
*Personal*				
Satisfaction with life	COPSOQ	5	*In most ways my life is close to my ideal*	scale 0–100, high = positive
Positive thinking/ professional attitude/dissociation/ hobbies/ sport/ religiosity/spirituality/ self-reflection/ self-care	pilot study	each case 1	*How much do the following help you to handle the workload?*	not/little helpful vs. quite/very helpful
Resilience	RS-13	13	*I can accept it when not all people like me*	scale 1–91, 13–66 = low, 67–72 = moderate and 73–91 = high resilience
**Health and Wellbeing**				
Self-rated health	COPSOQ	1	*If you evaluate the best conceivable state of health at 10 points and the worst at 0 points: how many points do you then give your present state of health?*	scale 0–100, high = positive
Burnout	COPSOQ	6	*How often do you feel emotionally exhausted?*	scale 0–100, high = negative
Intention to leave the profession	COPSOQ	1	*How often in the last 12 months have you thought about giving up your profession?*	scale 0–100, high = negative
Chronic back pain	RKI	1	*In the last 12 months, did you had almost daily back pain, which persisted 3 months or longer?*	yes, no, I don’t know
Depression	PHQ-2	2	*Over the last 2 weeks, how often have you been bothered by the following problems?*	score 0–6, score ≥ 3 major depressive disorder

The PHQ-2 is the short version of the Patient Health Questionnaire-9 (PHQ-9), which is a valid and reliable instrument to measure depression [41]. The present study used the German version of the PHQ-2 questionnaire to collect information on the frequency of anhedonia and depressed mood during the last 2 weeks [42]. The question is: ‘Over the last two weeks, how often have you been bothered by the following problems?’ and the two items are ‘little interest or pleasure in doing things’ and ‘feeling, down, depressed, or hopeless’ with the response options ‘not at all’, ‘several days’, ‘more than half the days’, and ‘nearly every day’. They are scored as 0, 1, 2 and 3, thus the PHQ-2 score can range from 0 to 6 (Table 2). The recommended cut off value of ≥3 was used to classify depressive disorder.

- Resilience Scale (RS-13) Questionnaire

The RS-13 questionnaire is the short 13-item version of the original 25-item Resilience Scale which was developed by Wagnild and Young (1993) [43]. The German version of the RS-13 was developed by Leppert and colleagues (2008) and measures resilience, i.e. the ability to successfully adapt to critical life situations, on a 7-point scale with answer categories ranging from I do not agree to I fully agree. These categories were transformed to a score ranging from 13 to 91 (Table 2). A score between 13 and 66 was defined as low, a score between 67 and 72 as moderate and a score between 73 and 91 as having high resilience [44].

- Question about back pain from the health survey conducted by the Robert Koch Institute

The question about back pain was selected from the health survey conducted by the Robert Koch Institute [45]. The question is: ‘In the last 12 months, did you had almost daily back pain, which persisted 3 months or longer?’ with three answer categories (yes, no, I don’t know).

- Other parameters

To assess palliative care specific working conditions, the questionnaire was extended by further questions, which were based on qualitative interviews with experts from palliative care [46] and a cross-sectional pilot study conducted in the specialized palliative care in Rhineland-Palatinate in Germany [16, 30]. Questions regarding ‘burden due to organisational framework conditions’, ‘emotional burden due to death’, ‘burden due to care of patients’, ‘burden due to nursing care’, ‘burden due to care of relatives’ as well as questions regarding the resource ‘good working team’ were summarized to scales. The scale ‘burden due to organisational framework conditions’ consisted of 7 items and were collected with a five-point Likert scale (‘no burden’, ‘very low burden’, ‘low burden’, ‘high burden’, ‘very high burden’). The scales ‘emotional burden due to death’, ‘burden due to care of patients’, ‘burden due to nursing care’ and ‘burden due to care of relatives’ consisted of several items and were collected with a six-point Likert scale (‘does not apply’, ‘no burden’, ‘very low burden’, ‘low burden’, ‘high burden’, ‘very high burden’). The scale ‚good working team’ consisted of 4 items and was collected with a 4-point Likert scale (categories ranging from disagree to agree) (Table 2). The self-developed items of the scales were prepared according to the COPSOQ guidelines. The answer category ‘does not apply’ was assessed as ‘no burden’. Furthermore, single categorical questions to resources were added, which showed to be of crucial importance within the pilot study [16, 30]. The categorical variables regarding resources were dichotomized (example: not helpful/little helpful vs. quite helpful/very helpful).

Table 2 provides an overview of the themes and sources of questions, as well as examples for the questions and variable outcomes.

### Statistical analysis

All scales (COPSOQ and self-developed) were prepared according to the COPSOQ guidelines. Scale values were computed as the average of the values of the single items of a person, if at least half of the single items were answered [47]. The proportion of missing values for the single items of the scales was below 2% in SPC and below 3% in GPC. Scale values are presented as mean values. To assess the internal consistency of the scales, the Cronbach’s Alpha was used. Values > 0.7 were regarded as acceptable [48]. Descriptive statistics (absolute and relative frequencies, means, standard deviations (SD)) were used to describe the data. The independent samples t-test was used to compare the mean scale values of GPC and SPC nurses. Further, a difference of at least 5 points in the mean value of a scale demonstrates a relevant difference between groups, thus the mean values of the scales of nurses working in GPC and SPC were compared. This method is regularly used in COPSOQ studies because a difference of 5 points in the mean value represents a small to intermediate effect size [Cohen’s d] of 0.2–0.35 [49]. Only results being statistically significant and fulfilling the difference of at least 5 points in the mean value were interpreted as relevant differences. For comparisons between categorical variables, the chi-square test of homogeneity was used to determine whether observed sample frequencies in GPC and SPC differed significantly from expected frequencies. Effect sizes were computed (Phi for 2 × 2 contingency tables and Cramer’s V for larger tables), where values between 0.10 and 0.30 represents a small to medium effect size and values of 0.50 represents a large effect size [50]. The significance level was established at *p* <  0.05 (two-tailed).

Statistical analyses and graphical representation were performed using SPSS version 23.5 and Microsoft Excel 2016, respectively.

## Results

### Descriptive analyses

For GPC, 2982 questionnaires were sent out and 497 (16.7%) returned. For SPC, 3539 questionnaires were sent out and 1371 returned (38.7%). Due to low GPC participation in hospitals, these were excluded from the analysis (Table 1). After data cleaning, *n* = 437 nurses form the GPC and *n* = 1316 nurses from the SPC were included into further analyses.

### Characteristics of the study sample

A summary of the sample characteristics is given in Table 3. Nurses in SPC were little older than nurses in GPC (mean 46.1 vs. 42.8 years, *p* ≤ 0.001). Furthermore, GPC and SPC nurses differed in age structure, in particular in the lowest and highest age groups. More nurses in SPC reported higher rates of graduation and levels of education than nurses in GPC.

**Table 3:** Participant characteristics

	GPC (***n*** = 437)	SPC (***n*** = 1316)	Difference between GPC and SPC, p-value	Effect size
**Sociodemographic characteristics**				
Age grouped, no. (%)			≤ 0.001	0.161
< 30	76 (17.4)	109 (8.4)		
30–39	104 (24.4)	233 (18.1)		
40–49	92 (21.6)	366 (28.4)		
≥ 50	154 (36.2)	582 (45.1)		
Sex, no. (%)			0.201	
Female	388 (89.6)	1119 (87.3)		
Male	45 (10.4)	163 (12.7)		
**Education and Graduation**				
Education, no. (%)			≤ 0.001	0.202
Without school-leaving qualification/other qualification	11 (2.5)	16 (1.3)		
Secondary school leaving certificate	58 (13.4)	56 (4.4)		
Qualification intermediate school-leaving certificate	239 (55.3)	674 (52.5)		
Entrance qualification for studies at universities of applied sciences	73 (16.9)	216 (16.8)		
General qualification for university entrance	51 (11.8)	322 (25.1)		
Grade, no. (%)			≤ 0.001	0.521
Nursing/geriatric nursing assistant	79 (18.7)	196 (15.2)		
Nurse	75 (17.7)	835 (64.8)		
Geriatric nurse	196 (46.2)	136 (10.6)		
University diploma	10 (2.4)	96 (7.5)		
Others (in training, other education, no education)	64 (15.2)	25 (2.0)		
**Working specific aspects**				
Working area, no. (%)				
Nursing home	344 (78.7)			
Outpatient care	93 (21.3)			
Palliative care unit		441 (33.5)		
Hospice		538 (40.9)		
SAPV		337 (25.6)		
Professional experience grouped, no. (%)			≤ 0.001	0.275
0–15 years	259 (61.5)	401 (30.8)		
16–30 years	125 (29.7)	616 (47.4)		
31–50 years	37 (8.8)	283 (21.8)		
Professional experience in specialised palliative care in years, mean (SD)		6.5 (4.8)		
Additional qualification in palliative care			≤ 0.001	0.579
No	329 (76.2)	196 (14.9)		
Current qualification	17 (3.9)	69 (5.3)		
Yes	86 (19.9)	1049 (79.8)		
Extent of palliative care in percent, median (range)	20 (0–100)			
Death of patients grouped, no. (%)			≤ 0.001	0.487
0	62 (15.6)	45 (3.8)		
1–3	218 (54.9)	173 (14.6)		
≥ 4	117 (29.5)	965 (81.6)		
Exercise of nursing activities, no. (%)				
No		233 (17.9)		
Yes		1071 (82.1)		
Extent of employment, no. (%)			≤ 0.001	0.116
Part-time job	175 (40.4)	667 (53.7)		
Full-time job	258 (59.6)	575 (46.3)		
Fund, no. (%)			≤ 0.001	0.317
Private	189 (44.7)	209 (16.4)		
Publicly-owned	28 (6.6)	338 (26.4)		
Independent	206 (48.7)	731 (57.2)		

*Note.* Valid percentages, missing values GPC: age (*n* = 11), sex (*n* = 4), education (*n* = 5), grade (*n* = 13), professional experience (*n* = 16), additional qualification (*n* = 5), death of patients = 40; extent of palliative care = 121, extent of employment (*n* = 35), fund (*n* = 14), missing values SPC: age (*n* = 26), sex (*n* = 34), education (*n* = 32), grade (*n* = 28); professional experience (*n* = 16), professional experience in palliative care (*n* = 30), additional qualification (*n* = 2), death of patients = 133; exercise of nursing activities (*n* = 12); extent of employment (*n* = 74), fund (*n* = 38)

78.7% of nurses in GPC worked in nursing homes and 21.3% in outpatient care. 40.9% of nurses in SPC worked in hospices, 33.5% in palliative care units and 25.6% in SAPV institutions. SPC nurses had more professional experience. More SPC nurses reported an additional qualification in palliative care than GPC nurses. On average (median), GPC nurses reported spending 20% of their time in the care of palliative patients. SPC nurses experienced more deaths of patients in the last month than GPC nurses. 17.9% of SPC nurses served in an advisory function only, meaning that they did not engage in any practical nursing activities. More SPC nurses reported a part-time-job than GPC nurses and more SPC nurses worked in health facility with publicity-owned or independent fund.

### Scales and single items

Table 4 presents the means and standard deviations of the scales reported in GPC and SPC. All scales achieved satisfactory values of internal consistency. Only the scale ‘workplace commitment’ in the field of SPC had a Cronbach’s Alpha of 0.677. Furthermore, it is specified whether a difference in the mean value of scales is given between GPC and SPC. In order to achieve transparency, we show the distribution of the response items of each self-developed scale for GPC and SPC in the supplementary material (see additional Figures 1, 2, 3, 4, 5 and 6). Table 5 presents results of single items regarding resources.

**Table 4:** Scales – Means, standard deviations and differences between GPC and SPC

Scales	GPC		SPC		Difference in mean	***p***-value	Difference ≥ 5
Burdens	M	SD	M	SD			
Burden due to organisational framework conditions	46.5	21.2	45.0	19.8	1.5	0.180	no
Quantitative demands	55.4	20.8	42.7	18.5	12.7	< 0.001	yes
Emotional demands	58.6	20.1	65.6	17.9	7.0	< 0.001	yes
Demands for hiding emotions	41.4	26.7	36.5	21.3	4.9	0.001	no
Emotional burden due to death	48.2	27.0	48.3	20.6	0.1	0.976	no
Burden due to care of patients	49.9	22.7	49.1	20.5	0.8	0.516	no
Burden due to nursing care	47.6	23.2	57.5	20.8	9.9	< 0.001	yes
Burden due to care of relatives	44.5	23.3	50.6	19.2	6.1	< 0.001	yes
**Resources**							
Meaning of work	82.2	18.8	88.3	13.3	6.1	< 0.001	yes
Workplace commitment	56.3	22.0	60.8	18.7	4.5	< 0.001	no
Good working team	70.5	22.5	77.7	18.0	7.2	< 0.001	yes
Satisfaction with life	66.9	19.4	71.5	17.4	4.6	< 0.001	no
**Health and Wellbeing**							
Self-rated health	61.9	19.7	72.9	16.9	11.0	< 0.001	yes
Burnout	48.8	20.3	41.4	17.6	7.4	< 0.001	yes
Intention to leave the profession	20.7	24.9	12.9	19.2	7.8	< 0.001	yes

*Note. M* Mean, *SD* Standard deviation

**Table 5:** Answers to single items – Resources and recognition

	GPC	SPC				
	%	%	Difference in %	X^2^-statistics	*p*-value	Effect size
Resource is quite/very helpful						
Religiosity/spirituality	25.5	47.0	21.5	60.997	< 0.001	0.188
Meaningfulness of work	77.1	91.5	14.4	63.527	< 0.001	0.191
Self-reflection	72.4	86.7	14.3	46.043	< 0.001	0.164
Sport	44.2	57.7	13.5	23.469	< 0.001	0.117
Self-care	76.4	86.5	10.1	24.256	< 0.001	0.119
Hobbies	77.5	86.8	9.3	21.034	< 0.001	0.111
Prof. attitude/dissociation	81.3	90.6	9.3	26.967	< 0.001	0.125
Gratitude of relatives	85.4	92.7	7.3	20.657	< 0.001	0.109
Positive thinking	82.2	88.3	6.1	10.344	0.001	0.078
Friends	84.3	87.4	3.1	2.655	0.103	
Gratitude of patients	90.5	92.5	2.0	1.797	0.180	
Family	87.4	87.8	0.4	0.057	0.812	
Recognition gained through						
Patients/relatives	84.7	98.3	13.6	125.515	< 0.001	0.269
Colleagues	81.9	90.9	9.0	25.905	< 0.001	0.122
Supervisor	61.8	68.0	6.2	5.406	0.020	0.056
Social context	83.6	89.3	5.7	9.712	0.002	0.075
Salary	30.0	26.7	3.3	1.757	0.185	

### Burdens

GPC nurses reported higher values on the quantitative demands scale than SPC nurses, whereas SPC nurses reported higher values on the emotional demands scale, higher values on the burden due to nursing care scale and higher values on the burden due to care of relatives scale. The highest difference in mean values concerned the quantitative demands scale. The lowest difference was assessed regarding the emotional burden due to death scale, where GPC and SPC nurses gave nearly the same results (Table 4).

### Resources

SPC nurses reported higher values on the meaning of work scale and the good working team scale than GPC nurses (Table 4). Furthermore, 27.5% of GPC nurses had low resilience, 17.2% had moderate resilience and 55.3% had high resilience. In SPC, 28.8% of nurses had low resilience, 21.3% had moderate and 49.9% had high resilience. Regarding the latter, there was no significant difference between GPC and SPC nurses.

Table 5 presents the frequency of resources mentioned by GPC and SPC nurses according the difference in the frequency of being mentioned. SPC nurses reported significantly more often religiosity and spirituality, meaningfulness of work, self-reflection, sport, self-care, hobbies, professional attitude/dissociation, gratitude of relatives and positive thinking as being helpful in dealing with the demands of their work than GPC nurses. Additionally, Table 5 presents the proportion of nurses agreed to having gained recognition through, which were reported by the nurses in both fields. SPC nurses reported significantly more frequently gained recognition from social contexts, from supervisors, from colleagues and from patients/relatives than GPC nurses.

### Health and wellbeing

Regarding health, on average nurses in GPC scored lower on the self-rated health scale and higher on the burnout scale than SPC nurses (Table 4). 52.1% of GPC nurses and 38.3% of SPC nurses reported chronic back pain. 3.2% of SPC nurses and 11.5% of GPC nurses exceeded the cut-off value of 3, where a major depressive disorder is likely. Both, chronic back pain as well as a major depressive disorder, were reported more frequently from nurses of GPC (chronic back pain: difference 13.8%, x^2^(1) = 25.098, *p* < 0.001, Phi = 0.121; major depressive disorder: difference 8.3%, x^2^(1) = 43.044, *p* < 0.001, Phi = 0.159). Further, GPC nurses reported a higher value on the intention to leave the profession scale (Table 4).

### Summary

Table 6 presents a summary of the items analysed in this study. In 4 of 8 reported burdens there was no difference (Table 6 ↔) between nurses in GPC and SPC. SPC nurses reported higher values three times and GPC nurses reported higher values once (Table 6 ↑). Concerning resources, in 7 out of 22 items, there was no significant difference (Table 6 ↔). In 15 items, SPC nurses reported significantly more often resources that were helpful in dealing with the demands of the work (Table 6 ↑). Regarding health and wellbeing, SPC nurses reported a better self-rated health (Table 6 ↑) and GPC nurses reported chronic back pain more often, higher values on the burnout scale and the intention to leave the profession more often (Table 6 ↑). Furthermore, a major depressive disorder is more likely in GPC nurses (Table 6↑).

**Table 6:** Summary – Comparison of the burdens, resources, health and wellbeing of nurses in GPC and SPC

			Variable	GPC		SPC
**Burdens**		Burden due to organisational framework conditions	scale		↔	
		Quantitative demands	scale	↑		
		Emotional demands	scale			↑
		Demands for hiding emotions	scale		↔	
		Emotional burden due to death	scale		↔	
		Burden due to care of patients	scale		↔	
		Burden due to nursing care	scale			↑
		Burden due to care of relatives	scale			↑
**Resources**	*Organisational*	Meaning of work	scale			↑
		Workplace commitment	scale		↔	
		Meaningfulness of work	single item			↑
		Gratitude of patients	single item		↔	
		Gratitude of relatives	single item			↑
		Recognition through supervisor	single item			↑
		Recognition through colleagues	single item			↑
		Recognition through patients/relatives	single item			↑
		Recognition through social context	single item			↑
		Recognition through salary	single item		↔	
	*Social*	Good working team	scale			↑
		Family	single item		↔	
		Friends	single item		↔	
	*Personal*	Satisfaction with life	scale		↔	
		Positive thinking	single item			↑
		Professional attitude/dissociation	single item			↑
		Hobbies	single item			↑
		Sport	single item			↑
		Religiosity/spirituality	single item			↑
		Self-reflection	single item			↑
		Self-care	single item			↑
		Resilience	single item		↔	
**Health and Wellbeing**		Self-rated health	scale			↑
		Burnout	scale	↑		
		Intention to leave the profession	scale	↑		
		Chronic back pain	item	↑		
		Depression	single item	↑		

*Note.* ↔ observed sample frequencies in GPC and SPC not differed significantly from expected frequencies or scale difference < 5; ↑ observed sample frequencies in GPC and SPC differed significantly from expected frequencies or scale difference ≥ 5 (*p* < 0.05)

## Discussion

The aim of the present study was to identify and compare the burdens, resources, health and wellbeing of nurses working in GPC and SPC in Germany. The key points of this comparison can be summarized as follows: First of all, nurses working in GPC and SPC showed differences in sociodemographic data as well as professional aspects. Secondly, SPC nurses reported higher emotional demands as well as higher burdens due to nursing care and the care of relatives while GPC nurses stated higher quantitative demands. Thirdly, SPC nurses reported more often resources that were helpful in dealing with the demands of their work and fourthly, SPC nurses stated a better health status and a lower intention to leave the profession than GPC nurses.

### Sociodemographic characteristics

SPC nurses were comparatively older than GPC nurses and reported higher professional experience. However, the relationship between age, professional experience and job related factors and health is not clear in the scientific discussion [51]. There were studies which revealed that high age has a negative effect on the health status [52] or work ability [53] of nurses. A study conducted in the field of end of life care found higher burnout scores in younger nurses with less professional experience. The authors of this study assumed that the obligation to be empathically available for patients and families as well as a lack of preparation in communication and work overload may make younger nurses or nurses with less professional experience more apprehensive, anxious and afraid of making mistakes [54]. Furthermore, there are studies which assessed no correlations [55] and studies which assessed diverse connections between different age groups and job-related factors and health [56]. The latter also has to be considered when further implications for future projects are made. An analysis based on data of the nurses’ early exit study (NEXT study) showed that older nurses had a worse state of health than younger nurses and that for younger nurses, leadership quality seemed to be an important component for preservation of a good health status. For older nurses, a good collaboration with the supervisor was important [52].

In connection with professional experience, a further aspect has to be considered. In the present study SPC nurses reported higher graduation levels and degrees of education and additional qualification in palliative care than GPC nurses. In Germany, this additional qualification, which covers an 160 h course in palliative care, is not obligatory for all SPC nurses. According to recommendations of the National Association of Statutory Health Insurance, all nurses in SAPV institutions should have an additional qualification to invoice for palliative care services from health insurance companies [57]. Palliative care experts from around the world considered the education and training of all staff in the fundamentals of palliative care to be essential [58]. A study conducted in Italy revealed that professional competency of palliative care nurses was positively associated with job satisfaction [59]. We assessed a positive effect of the additional qualification within the pilot study in SPC in relation to organisational demands and demands regarding the care of relatives [16]. In future studies, it is therefore necessary to consider whether and to what extent an additional qualification should be required for all nurses in palliative care.

SPC nurses reported having a full-time job respectively working in institutions with a private fund less often than GPC nurses. This may have an impact on their working conditions, their health and well-being. Regarding nursing homes in Germany, a study reported the highest burden in publicly-owned nursing homes and the lowest in independent nursing homes [60].

### Demands

SPC nurses reported higher emotional demands, greater burdens due to nursing care and greater burdens due to the care of relatives, while GPC nurses reported higher quantitative demands. Quantitative demands are elements of the work environment, related to the amount and the time conditions of work to be done [61]. Among the different subscales of burdens, the quantitative demands scale showed the greatest difference in the reported scale mean. SPC nurses reported a comparatively lower value (M = 42.7 vs. M = 55.4). COPSOQ reference data presents mean values of the scale ranging from M = 50.4 for occupations in the health sector [62], M = 51 for geriatric nurses, and M = 60 for nurses [63]. The relatively low mean value of the SPC nurses can be explained by the fact that SPC nurses, e.g. in palliative care units in Germany have fewer patients to care for than nurses in other fields [64] and that 18% of SPC nurses did not engage in any practical nursing activities. For years, minimum legal standards for the nurse-to-patient-ratio in Germany have been discussed. The Registered Nurse Forecasting Study (RN4CAST), one of the largest nurse workforce studies conducted in Europe in which 12 countries participated revealed, that the average ratio of patients to nurses across hospitals ranged from 5 in Norway, over 7 in the Netherlands to 13 in Germany [65]. The Federal Government in Germany has underlined current efforts with the Nursing Workforce Strengthening Act (Pflegepersonal-Stärkungs-Gesetz (PpSG)) [66] to improve working conditions for nursing care in hospitals and nursing homes for example regarding personnel requirements. The greater emotional demands and greater burdens due to care of relatives of SPC nurses can be explained by the different structures and aims of the health and palliative care institutions. SPC nurses care for patients whose nursing care is more complicated. Further, SPC not only concentrate on the patient but also on the families. A fact which has to be considered in evaluating the working situation of nurses in palliative care, and which was long disregarded in the research [16]. The burden due to nursing care scale which was used in the present study describes the most stressful nursing care situations of SPC nurses, which were reported in the pilot study [16, 30, 46]. This can explain why the burden due to nursing care in this study is higher for nurses in SPC. Consequently, various other possible demands of nurses, particular of nurses working in GPC, were not collected. In order to gain a deeper understanding on how the level of nursing care differs between GPC and SPC nurses, further research is needed.

The lowest difference between nurses in GPC and SPC was identified regarding emotional burden due to death. This was surprisingly, because nurses in SPC reported many more deaths of patients than nurses in GPC. Nevertheless, the experienced burden was nearly the same. There are different possible explanations for this aspect. Firstly, SPC nurses were comparatively older, had more professional experience and reported an additional qualification in palliative care more frequently than nurses in GPC, as already discussed. Secondly, nurses in SPC stated more frequent that they have various resources which were helpful in dealing with the demands of their work. Thirdly, death and dying is demanding, but other factors play a more important role. In the pilot study, nurses in SPC reported that the death and dying is not of crucial importance for the perceived burden, but rather weather the patient received good care or not [16]. Finally, working in SPC is an active choice made by every nurse. Nurses are aware of the demanding care for palliative care patients and their families, but this care also seems to be enormously rewarding [67].

### Resources

SPC nurses reported more often organisational, social and personal resources than GPC nurses. No differences were found according to workplace commitment, gratitude of patients, recognition of salary, family, friends and the personal resources satisfaction with life and resilience. The highest differences in the frequencies were assessed regarding meaningfulness of work, recognition through patients and relatives, a good working team, religiosity/spirituality, self-reflection, sport and self-care. Self-care is broadly defined as self-initiated behaviour that people choose to incorporate and promote good health and general well-being into everyday life [68]. Further, it is about being healthy but also about incorporating coping strategies in life to deal with work stressors. Self-care can sustain well-being and resilience [23, 69]. The importance of self-care is deeply rooted in SPC. Self-care trainings [70] or self-care plans [71] are offered in SPC. Particularly with regard to the COVID-19 pandemic, self-care and self-care trainings for healthcare workers become important [72–74].

Various studies identified the team as an essential resource in the field of palliative care [16, 18, 75, 76] or support from co-workers and supervisors in the nurse setting [77]. Recognition through patients and relatives was already described as a key element in creating and sustaining healthy work environments [78]. The American Association of Critical-Care Nurses (AACN) published AACN Standards for Establishing and Sustaining Healthy Work Environments, in which meaningful recognition represents one from six standards needed to create a healthy work environment [79]. Although a study which concentrated on the work motivation of nurses assessed that extrinsic rewards such as payment, promotion and fringe benefits were the basic sources of motivation and intrinsic reward, such as recognition, appeared to be less important [80]. Noticeable in this context is that the majority of nurses in both GPC and SPC do not feel to gain recognition through their salary. In Germany, the Federal Government underling current efforts with the already mentioned Nursing Workforce Strengthening Act (Pflegepersonal-Stärkungs-Gesetz (PpSG), which also affects higher salaries for nurses. As our study indicates, future efforts should concentrate on the balance of extrinsic and intrinsic rewards in the nurse setting, in order to achieve the best balance to promote the health and satisfaction of nurses.

### Health and wellbeing

GPC nurses stated in all elevated aspects worse values than SPC nurses. They reported a worse self-rated health, higher burnout levels, more frequent chronic back pain, more frequent major depressive disorders and greater intention to leave the profession. Regarding the latter, SPC nurses reported a lower value on the scale (M = 12.9), other studies from Germany reported higher values (M = 19 (t1) and M = 15 (t2) [52], M = 18 [81] and GPC nurses reported the highest value (M = 20.7). The results relating to burnout matches the results of a recent published review where healthcare professionals in GPC experience more symptoms of burnout than those in specialised palliative care settings [35].

In the light of demographic developments, future analysis of the data is needed to find out why SPC nurses seems to be more satisfied with their work than GPC nurses and which impact the single burdens and resources have, not only on job satisfaction but also on health.

## Limitations

The results of the comparison of the working situation of nurses working in GPC and SPC in Germany must be interpreted with caution due to the different structures and aims of the health and palliative care institutions [7, 11]. Additionally, the present study compared the data of GPC nurses, which represent the merged data of nurses working in nursing homes and outpatient care, with the data of SPC nurses, which represent the merged data of nurses working in SAPV institutions, hospices and palliative care units. A great deal of information thus gets lost because the comparison is built on a macro level, the social structures of care. In the future, comparisons of nurses on a meso level in single areas and institutions will follow, but this was not part of this paper. The survey instrument of the present study included mostly valid and reliable instruments, such as the COPSOQ. Furthermore, it included additional self-developed questions. The latter were not validated but were valuable for our study as they answered certain questions that standardized questionnaires could not. It should be noted that the self-developed scales were developed to address palliative care specific working conditions of nurses focusing on the working conditions in SPC. Consequently, various other possible demands of nurses working in GPC were not collected. In order to achieve transparency, we showed the frequency of the response items of each self-developed scale for both areas. Further, the present study focused firstly on medical facilities. Only the participating facilities reported the number of staff members. The low participation and response rate of GPC nurses raises the possibility of selection bias. Although a random sample was drawn, this sample was not representative of GPC due to different response behaviours and the exclusion of hospitals. The lower response rate of GPC could be responsible for the differences between the burdens, resources and health status between GPC and SPC. A comparison with participants and no participants of this survey was whether within GPC nor SPC feasible. It should be noted that it is likely that nurses who experience greater burdens were less motivated to respond to a time-consuming survey. Therefore, it is possible that the demands in the present study were underestimated. Additionally, because of the two samples, only exploratory and no confirmatory data analysis was possible and the results presented are based on comparisons of means and sample frequencies. The cross-sectional design of the study cannot prove causality between burdens, resources, health and wellbeing. Therefore, interventional and longitudinal studies at the micro level in nursing practice are needed to support causality in the relationships of burdens, resources, health and wellbeing.

## Conclusions

This is the first nationwide study in Germany to compare the working situations of GPC and SPC nurses in various settings providing a large amount of information. Overall, the working situation of GPC and SPC nurses were different and the nurses reported burdens in several working areas. However, the study demonstrated that although nurses in SPC overall reported a higher level of burden than those in GPC, SPC nurses stated that they had a better health status and a lower intention to leave the profession than GPC nurses. Further, SPC nurses differenced in the frequency of reported resources, which were helpful in dealing with the demands of their work to GPC nurses. The results of the present study may be used to develop individual concepts for improving health and wellbeing of nurses taking the differences between GPC and SPC into account. While SPC nurses for example often reported self-care as a resource, future interventions in the field of GPC could take self-care as a subject of discussion into account [82].

In the future, the demographic differences, further participants’ characteristics as well as the differences in the burdens and resources should be further analysed in order to examine which have the biggest impact on health status and intentions of leaving the profession. Additionally, future studies should review SPC as the best practice example for nursing care in Germany.

The implementation of palliative care differs strongly around Europe [6] and around the world [68]. Future research is needed in order to find out to what degree the presented results can be transferred to other countries. Nevertheless, the results of the present study could have relevant implications for developing interventional studies, with the goal of improving the health status of nurses and enhancing job satisfaction. This includes first of all an improvement of working conditions like personal requirements, but simultaneously the strengthening of organisational, social and personal resources.

## Supplementary Information

**Additional file 1: Additional Table****1****.** Palliative care in Germany.

**Additional file 2: Additional Figure 1.** Burden due to organisational framework conditions (GPC: *n* = 437, SPC: *n* = 1316). **Additional Figure 2.** Emotional burden due to death (GPC: *n* = 437, SPC: *n* = 1316). **Additional Figure 3.** Burden due to care of patients (GPC: *n* = 437, SPC: *n* = 1316). **Additional Figure 4.** Burden due to nursing care (GPC: *n* = 437, SPC: *n* = 1316). **Additional Figure 5.** Burden due to care of relatives (GPC: *n* = 437, SPC: *n* = 1316). **Additional Figure 6.** Good working team (GPC: *n* = 437, SPC: *n* = 1316).

## Data Availability

The whole data set is available at the University Medical Centre of the University of Mainz, Department of Occupational, Social and Environmental Medicine. Contact: elidiehl@uni-mainz.de

## Abbreviations

GPC: General Palliative Care
SPC: Specialised Palliative Care
COPSOQ: Copenhagen Psychosocial Questionnaire
PHQ-2: Patient-Health Questionnaire
RS-13: Resilience questionnaire-13
EAPC: European Association for Palliative Care
SAPV: Specialist outpatient palliative care services
M: Mean
SD: Standard deviation
