# Supplementary material for: Burdens, resources, health and wellbeing of nurses working in general and specialised palliative care in Germany – results of a nationwide cross-sectional survey study
Source: BMC Nurs. 2021 Sep 6;20:162. doi: 10.1186/s12912-021-00687-z (PMC8419389; doi:10.1186/s12912-021-00687-z)
Supplement: Supplementary file 1 — Additional file 1: Additional Table1. Palliative care in Germany. [file 12912_2021_687_MOESM1_ESM.docx]

Additional Table 1: Palliative Care in Germany

|  | **General Palliative Care (GPC)** | **Specialised Palliative Care (SPC)** |
| --- | --- | --- |
| **Outpatient** | - Outpatient care | - Specialist outpatient palliative care (SAPV) |
| **Inpatient** | - Hospitals - Nursing homes | - Palliative care units in hospitals - Inpatient hospices* |

*Note.* * Independent facilities which are mostly allocated to SPC. Furthermore there are outpatient hospice services (support is provided by volunteer staf), hospital palliative care support teams or palliative care networks which provide palliative care. References: Deutsche Gesellschaft für Palliativmedizin. Definitionen zur Hospiz- und Palliativversorgung. https://www.dgpalliativmedizin.de/images/DGP_GLOSSAR.pdf (accessed on 12 February 2020); Deutscher Hospiz- und PalliativVerband e.V. Hospizarbeit und Palliativversorgung. <https://www.dhpv.de/themen_hospiz-palliativ.html> (accessed on 20 May 2020); Nauck, F. Sterbehilfe - Streit um eine gesetzliche Neuregelung: Palliative Versorgung zu Hause und in der Klinik. Status quo und Perspektiven. *Frankfurter Forum: DISKURSE* 2015, 1, 12–21; German National Academy of Sciences Leopoldina and Union of German Academies of Sciences. *Palliative care in Germany*. *Perspectives for practice and research*; Monograph series on science-based policy advice, 2015. http://nbn-resolving.de/urn:nbn:de:gbv:3:2-62819.
