# Supplementary material for: Burdens, resources, health and wellbeing of nurses working in general and specialised palliative care in Germany – results of a nationwide cross-sectional survey study
Source: BMC Nurs. 2021 Sep 6;20:162. doi: 10.1186/s12912-021-00687-z (PMC8419389; doi:10.1186/s12912-021-00687-z)
Supplement: Supplementary file 2 — Additional file 2: Additional Figure 1. Burden due to organisational framework conditions (GPC: n = 437, SPC: n = 1316). Additional Figure 2. Emotional burden due to death (GPC: n = 437, SPC: n = 1316). Additional Figure 3. Burden due to care of patients (GPC: n = 437, SPC: n = 1316). Additional Figure 4. Burden due to nursing care (GPC: n = 437, SPC: n = 1316). Additional Figure 5. Burden due to care of relatives (GPC: n = 437, SPC: n = 1316). Additional Figure 6. Good working team (GPC: n = 437, SPC: n = 1316). [file 12912_2021_687_MOESM2_ESM.docx]

Additional Figure 1: Burden due to organisational framework conditions (GPC: n = 437, SPC: n = 1,316)

Additional Figure 2: Emotional burden due to death (GPC: n = 437, SPC: n = 1,316)

Additional Figure 3: Burden due to care of patients (GPC: n = 437, SPC: n = 1,316)

Additional Figure 4: Burden due to nursing care (GPC: n = 437, SPC: n = 1,316)

Additional Figure 5: Burden due to care of relatives (GPC: n = 437, SPC: n = 1,316)

Additional Figure 6: Good working team (GPC: n = 437, SPC: n = 1,316)
